# 1D Piezoelectric Material Based Nanogenerators: Methods, Materials and Property Optimization

**DOI:** 10.3390/nano8040188

**Published:** 2018-03-23

**Authors:** Xing Li, Mei Sun, Xianlong Wei, Chongxin Shan, Qing Chen

**Affiliations:** 1 Department of Physics and Engineering, Zhengzhou University, Daxue Road 75, Zhengzhou 450052, China; xingli@zzu.edu.cn (X.L.); cxshan@zzu.edu.cn (C.S.); 2 Key Laboratory for the Physics and Chemistry of Nanodevices and Department of Electronics, Peking University, Beijing 100871, China; sunmei@pku.edu.cn (M.S.); weixl@pku.edu.cn (X.W.)

**Keywords:** 1D piezoelectric materials, characterization methods, piezoelectric property optimization, size effect, structure and orientation dependence, defects

## Abstract

Due to the enhanced piezoelectric properties, excellent mechanical properties and tunable electric properties, one-dimensional (1D) piezoelectric materials have shown their promising applications in nanogenerators (NG), sensors, actuators, electronic devices etc. To present a clear view about 1D piezoelectric materials, this review mainly focuses on the characterization and optimization of the piezoelectric properties of 1D nanomaterials, including semiconducting nanowires (NWs) with wurtzite and/or zinc blend phases, perovskite NWs and 1D polymers. Specifically, the piezoelectric coefficients, performance of single NW-based NG and structure-dependent electromechanical properties of 1D nanostructured materials can be respectively investigated through piezoresponse force microscopy, atomic force microscopy and the in-situ scanning/transmission electron microcopy. Along with the introduction of the mechanism and piezoelectric properties of 1D semiconductor, perovskite materials and polymers, their performance improvement strategies are summarized from the view of microstructures, including size-effect, crystal structure, orientation and defects. Finally, the extension of 1D piezoelectric materials in field effect transistors and optoelectronic devices are simply introduced.

## 1. Introduction

To alleviate the severe energy problems we are facing nowadays, tremendous attention has been paid on harvesting clean and renewable energy from ambient energy sources. Given the enhanced piezoelectric effect and excellent mechanical properties, one-dimensional (1D) piezoelectric nanostructures have been regarded as the next-generation piezoelectric material. Meanwhile, during the miniaturization of various functional devices, their high specific surface area, low energy consumption and easier integration [1,2,3,4] also made them promising building blocks for future electronic devices, where the development of wireless and self-powered electronic devices are quite essential, especially in the field of sensing, medical science and wearable personal electronics. To self-power these nanodevices, the development of nanogenerators (NGs) harvesting energy from the environment is indispensable.

In order to harvest different forms of energy from environment, various types of NGs have been recently reported by utilizing the effects of piezoelectric [5], triboelectric [6,7,8,9,10,11], pyroelectric [12,13], thermoelectric [14] and ion streams [15,16] etc. Pyroelectric NGs and thermoelectric NGs are designed to convert heat energy into electricity by utilizing the time-dependent temperature change or temperature gradient existing in the devices. The microfluidic generator has been demonstrated to convert the hydroenergy of fluid into continuous electrical output with the streaming potential. To harvest the abundant mechanical energy in the ambient environment, the triboelectric NG (TENG) and piezoelectric NG (PENG) have been presented. TENG is generally invented based on the coupling of triboelectrification and electrostatic induction to convert the mechanical friction energy to electricity [17]. By utilizing the strain-induced piezoelectric polarization, the performance of PENG relies greatly on the piezoelectric properties of materials. Since the piezoelectric properties can in turn influence the electronic/optoelectronic properties of certain materials, their applications have been further extended in piezoelectronics and piezo-phototronics.

Expect for the energy harvesting applications, mechanical strain has also been utilized to mediate the transport properties of semiconductors and shown great potential in applications utilizing strain induced properties change. The piezoresistive effect has been widely introduced into field effect transistors (FETs) to increase their carrier mobilities [18,19]. Different from the piezoresistive effect which is related to the strain induced band structure change, the piezoelectric effect is caused by the relative displacement of cations and anions under strain in non-central symmetric crystal structures. For piezoelectric semiconducting nanowires (NWs), the simultaneous possession of piezoelectricity and semiconductivity makes it possible to control the charge carrier transport in electronic devices and the carrier generation, transport, separation and recombination in the optoelectronic devices with strain induced piezo-potential [20]. Therefore, the exploration of piezoelectric semiconducting NWs is also very important to further improve their performance and extend their applications. Since Wang and Song’s pioneering work on the first NG using piezoelectric semiconducting ZnO NWs [5], various characterization methods for the piezoelectric properties of 1D nanostructures have been developed. Up to now, various piezoelectric semiconducting NWs like CdS, GaN, ZnS, InN and InAs etc. [21,22,23,24] have been studied and the corresponding NGs, strain-gated transistors and switches have been demonstrated [25,26]. Moreover, the performance of solar cells [27], photodetectors [20] and light-emitting diodes [28] based on these materials have been remarkably improved by the piezo-phototronic effect.

During the past decades, various 1D nanostructures with different size, structure, chemical composition and doping [29,30] have been successfully synthesized and their applications in mechanical, electromechanical, electric and optoelectronic devices have been demonstrated [31,32,33]. Importantly, it is desirable to know which set of NW morphological (diameter, length), structural (crystal structure, defect type and density, etc.), and electrical properties (conductivity, polarizability) gives the best performance for a particular application. Therefore, clarification of these parameters to the piezoelectric properties of 1D nanomaterials is of vital importance for the performance optimization of PENG.

To present a clear view about 1D piezoelectric materials, this review will start with the characterization method of piezoelectricity in individual 1D nanomaterials. The piezoresponse force microscopy (PFM) method, lateral bending with atomic force microscopy (AFM) and the tensile loading accomplished by in-situ scanning electron microscopy (SEM) and transmission electron microscopy (TEM) are introduced in detail in Section 2. Then, the recently investigated 1D piezoelectric nanomaterials of semiconducting NWs, ferroelectric perovskite NWs, polymers and their corresponding piezoelectric properties are summarized in Section 3. In order to provide clear instructions for the performance optimization, we summarize the influence of crystal orientation, size and defects like doping and grain boundaries on the piezoelectric properties of 1D nanomaterials in Section 4. Finally, applications of the piezoelectric effect of these nanostructures in FETs and optoelectronic devices are simply introduced.

## 2. Characterization Methods of Piezoelectricity in One-Dimensional (1D) Nanomaterials

To evaluate the performance of piezoelectric materials, it is of vital importance to measure the piezoelectric coefficients, which quantify the volume change when a piezoelectric material is subject to an electric field. However, due to the nano-scale dimensions and displacement of 1D nanomaterials, traditional methods like the Berlincourt method and laser interferometry can hardly be applied to accurately measure their corresponding piezoelectric properties [34,35]. Therefore, the recently developed methods based on PFM, AFM and in-situ SEM/TEM in measuring the electromechanical properties of 1D NWs are firstly reviewed in this section.

### 2.1. Piezoresponse Force Microscopy Method: Piezoelectric Coefficient Measurement of Single Nanowire (NW)

PFM method is firstly applied by Zhao et al. in 2004 to measure the effective piezoelectric coefficient of ZnO nanobelt [36] and has been frequently used to investigate the piezoelectric response of LiNbO_3_ NWs [37], Sb_2_S_3_ NWs [38], perovskite nanofibers/NWs [39,40,41], GaN NWs [42] etc. PFM is a method based on the converse piezoelectric effect at the nanoscale which can be defined by:

(1) $$\varepsilon_{j}=d_{ij}E_{i}$$

where *ε_j_* is the strain, *d_ij_* is the piezoelectric coupling coefficient and *E_i_* is the applied electric field [43]. Specifically, the measurement is based on an AFM equipped with PFM mode. NWs are generally dispersed onto a substrate with a conductive layer, which is used as a bottom electrode. Taking advantage of the subnanoscale measurement capabilities of AFM, the local displacement of a sample induced by the applied electric field can be precisely detected.

**Figure 1:**
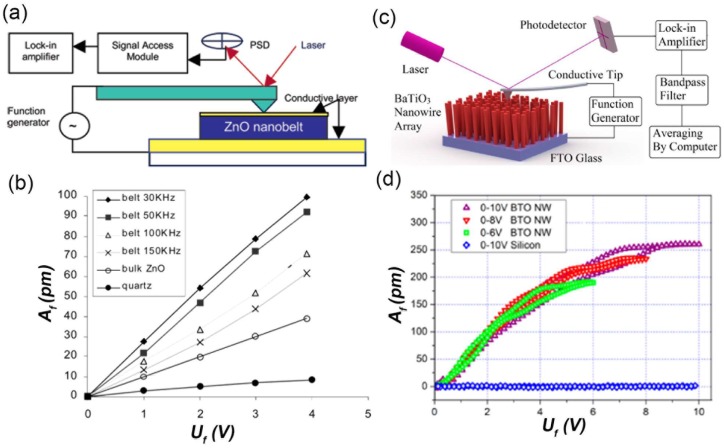
The piezoelectric coefficient measurement with the piezoresponse force microscopy (PFM) method. (**a**) The schematic diagram of experimental setup of the PFM method in measuring *d*_33_ along the radial direction of the lateral dispersed one dimensional (1D) nanostructures; (**b**) The *A_f_*-*U_f_* curve obtained with the PFM method, the piezoelectric coefficient can be obtained from the slope of the linear curve. Reproduced with permission from [36]. American Chemical Society, 2004; (**c**) The schematic diagram of the experimental setup of the refined PFM method in measuring *d*_33_ along the axial direction of the vertically grown nanowire (NW) array; (**d**) The *A_f_*-*U_f_* curve of a BaTiO_3_ NW with refined PFM method. Reproduced with permission from [41]. American Chemical Society, 2013.

Specifically, the measured NW should be firstly located with the tapping mode of AFM to avoid the displacement of NW during the tip scanning of the contact mode. Then, the AFM tip is positioned to the center of the located NW/nanofiber and is followed by switching to the contact mode. An intermediate force is applied to ensure that the tip deflection is dominated by the electromechanical response of the NW rather than electrostatic forces. Subsequently, an alternating current (AC) signal is applied between the conductive AFM tip and the bottom electrode. Meanwhile, the corresponding vertical deflection signal of the AFM cantilever is recorded by the lock-in amplifier through a signal access module (Figure 1a). Thus, the amplitude of the AFM tip vibration can be obtained by multiplying the deflection signal with the calibration constant of the photodetector sensitivity [44]. Finally, the effective piezoelectric coefficient *d*_33_ can be derived from the slope of *A_f_*-*U_f_* curve according to:

*A_f_* = *d*_33_*U_f_* (2)

where *A_f_* is the vibration amplitude and *U_f_* is the amplitude of the testing AC voltage (Figure 1b).

In the above configuration, the electric field and induced displacement are both along the radial direction of the 1D nanostructures. To demonstrate the piezoelectric behavior of BaTiO_3_ NWs in their longitude direction (axial direction), a refined PFM testing method is applied by Zhou et al. [41]. As shown in Figure 1c, vertically NW arrays grown on a conductive substrate is used. The electrical field is applied along its axial direction through the conductive AFM tip. A band pass filter is used to get the real displacement of the NW’s top surface. Finally, the piezoelectric coefficient of the BaTiO_3_ NWs in the longitude direction can be measured through the slope of the *A_f_*-*U_f_* curve as well (Figure 1d).

**Figure 2:**
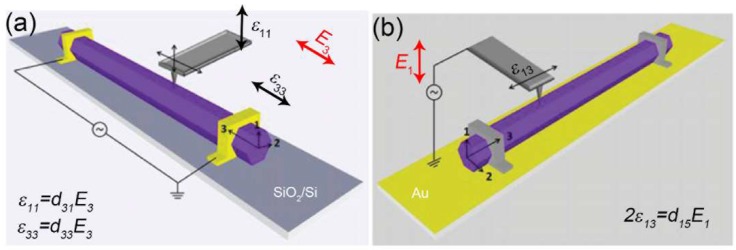
The schematic representations of measurement configurations for probing the piezoelectric coefficients of (**a**) *d*_33_, *d*_31_ and (**b**) *d*_15_ of a single *c*-axis GaN NW. Reproduced with permission from [23]. American Chemical Society, 2011.

Moreover, Minary-Jolandan et al. have presented an experimental approach to directly quantify the three independent piezoelectric coefficients (*d*_33_, *d*_31_ and *d*_15_) of individual wurtzite (WZ) NWs [23]. As shown in Figure 2a, the NW laying on Si substrate with an insulating SiO_2_ layer is clamped at two ends by metal contacts. During these measurements, it is critical to place the *c*-axis of the GaN NW perpendicular to the long axis of the AFM cantilever [42]. Similar to the aforementioned PFM method, to measure the piezoelectric constants *d*_31_ and *d*_33_, an AC voltage should be applied in the axial direction of the NW to create an axial electric field *E*_3_. Meanwhile, the axial (*ε*_33_) and out-of-plane displacement (*ε*_11_) can be measured through the twist and bending of the AFM cantilever. The *d*_15_ constant is obtained by applying a transverse electric field (*E*_1_) across the NW (Figure 2b) and measuring the shear strain (*ε*_13_) with the torsion of the cantilever. Bowland et al. also applied similar method to investigate the *d*_33_ and *d*_31_ piezoelectric coefficient of BaTiO_3_ coated carbon fibers [43].

Therefore, by taking advantage of AFM in accurately measuring nanoscale displacement, piezoelectric coefficients can be directly and systemically obtained by controlling the electric field direction and the displacement measurement direction.

### 2.2. Atomic Force Microscopy Lateral Bending: Performance Evaluation of Single NW-Based Nanogenerators (NGs)

With the aforementioned PFM method, the piezoelectric coefficients of 1D nanostructures can be successfully obtained on the basis of converse piezoelectric effect, which refers to electric field induced displacement of piezoelectric materials. To convert the mechanical energy into electric power (direct piezoelectric effect), the AFM bending method is subsequently demonstrated [5], which is generally performed by AFM using a conductive AFM tip with calibrated normal spring constants.

**Figure 3:**
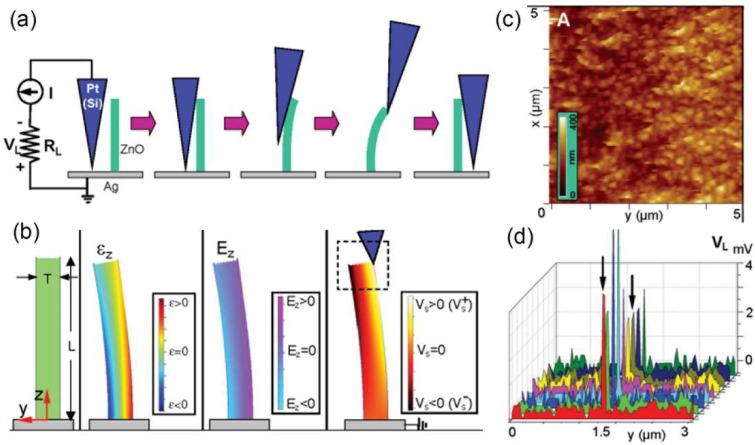
Piezoelectric nanogenerator (NG) investigated with the atomic force microscopy (AFM) lateral bending method. (**a**) Experimental setup and procedures for generating electricity by deforming a vertically grown ZnO NW with a conductive AFM tip; (**b**) Simulation results of the longitudinal strain, corresponding piezoelectric induced electric field and potential for a bended ZnO NW; (**c**) The topography image of the measured ZnO NW array; (**d**) A series of line profiles of the voltage output signal when the AFM tip scanned across a vertical NW at a time interval of 1 min. Reproduced with permission from [5]. The American Association for the Advancement of Science, 2006.

Generally, the electrical contact between the bottom end of the NW and measurement circuit should be firstly made. When the AFM tip is scanned over the sample in contact mode, the vertically grown NWs will be bent consecutively (Figure 3a) with the outer surface being stretched and the inner surface compressed. Therefore, a strain field will be created and a piezoelectric potential is consequently created in the NW due to the polarization of the ions (Figure 3b). Specifically, positive and negative piezoelectric potentials are distributed respectively along the stretched and compressed side of the tested NWs and the corresponding values depend on the magnitude of mechanical strains. Along with the bending of NWs, we can simultaneously record the topography (Figure 3c) and the corresponding output voltage *V_L_* across an outside resistance (Figure 3d), where the bending distance and voltage output values can be directly obtained.

The constant height (tip-substrate distance *Z*) mode has been considered more appropriate in controlling NW bending than the constant force mode and has been recently applied by Alekseev et al. to investigate the performance of GaAs NW based NG [45]. When the NW is scanned by an AFM tip with a distance *Z* smaller than the NW’s length *L*, bending of the NW will be introduced by the AFM tip. Reducing this distance leads to increased deformation of the NW. Thus, the dependency of electrical signal on the distances can be obtained. Compared with the PFM method, the AFM bending method shows its advantage in demonstrating the performance of NGs based on single NW. It should be noted that in this method, the contact between NW and the AFM tip should be Schottky contact. In such case, the bending-induced piezoelectric potential can be accumulated until it is higher than the contact barrier and subsequently release through a peak current. If the contact between the NW and the AFM tip is Ohmic contact, any small polarization of the ions will be immediately released through small current, no peak current can be observed. The AFM lateral bending method has been widely used to investigate the performance of CdS [21], InN [22], AlN [46] and GaN [47] NWs based PENGs.

### 2.3. In-Situ Scanning/Transmission Electron Microscopy Method: Microstructural Dependenct Performance of NW Based-NG

By introducing micromanipulators or multifunctional sample holders into SEM and TEM, the in-situ techniques have been widely applied to study the mechanical [48,49,50], electrical [51] and electrochemical properties [52] of various nanomaterials. Since the mechanical strain can be exerted with direct observations and the corresponding electrical response can be simultaneously measured, in-situ SEM/TEM methods have been widely used to study the electromechanical properties of 1D nanostructures. Moreover, these methods also show their advantage in revealing the influence of crystal structure, orientation, defect, size etc. on the piezoelectric properties.

**Figure 4:**
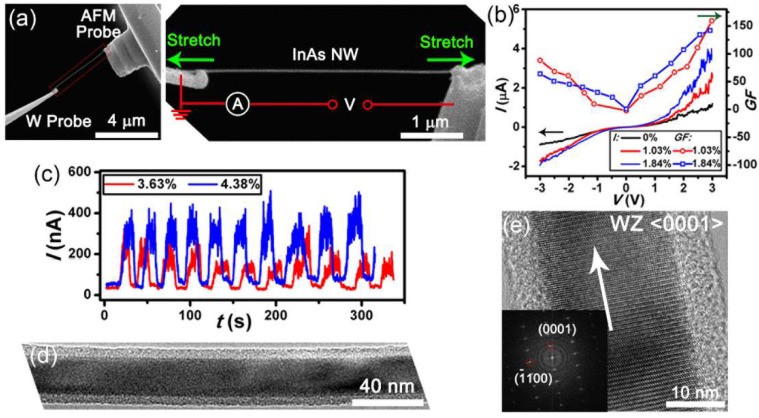
In-situ scanning electron microscopy (SEM) tensile test in measuring the piezoelectric and piezoresistive effects of InAs NWs. (**a**) SEM image showing the experimental setup for the electromechanical measurement of InAs NWs; (**b**) The measured current-voltage (*I*-*V)* and gauge factor-voltage (*GF*-*V)* curves and (**c**) the electrical current responses of InAs NWs; (**d**) The low-magnification and (**e**) high-resolution transmission electron microscope (TEM) images of the NW measured in (**b**). Reproduced with permission from [24]. John Wiley and Sons, 2015.

**Figure 5:**
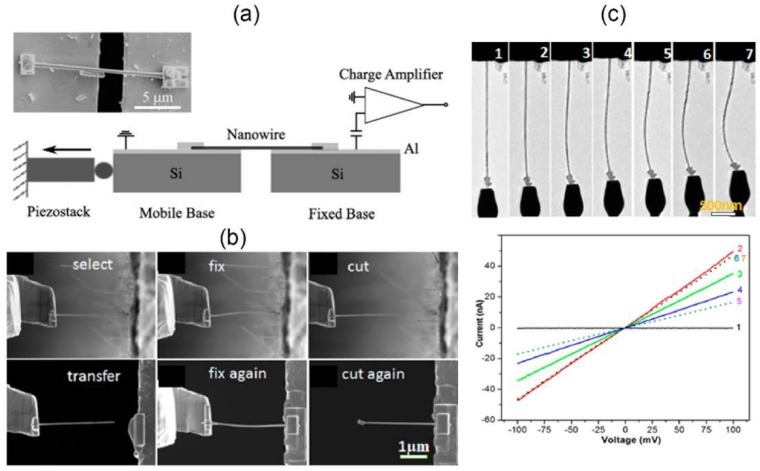
(**a**) The piezoelectric charge detection from an individual BaTiO_3_ NW with the tensile loading platform. Reproduced with permission from [54]. American Chemical Society, 2007; (**b**) SEM images showing the manipulation process for an individual InAs NW with a dual beam system; (**c**) TEM images showing the in-situ TEM deformation process of an InAs NW and corresponding *I*-*V* curves to each deformation state. Reproduced with permission from [56]. American Chemical Society, 2016.

Previously, we have investigated the crystal-structure-dependent piezoelectric and piezoresistive effects of InAs NWs with in-situ SEM tensile test [24]. Individual NW is firstly picked up from the substrate by micromanipulators and then is connected between two electrical probes with electron beam induced deposition of amorphous carbon (EBID) [53] (Figure 4a). Then, the NW is uniaxially stretched and its electrical transport properties are measured at different tensile strains (Figure 4b). The tensile strain of a stretched NW can be determined by:

(3) $$\varepsilon =\frac{L-L_{0}}{L_{0}}$$

where *L* and *L*_0_ are the NW length with and without axial stretching directly measured by SEM images. A conductive AFM probe with calibrated spring constant *k* can also be used here to measure the applied tensile stress. During the pulling process of individual NW, the corresponding electrical transport properties (Figure 4b,c) are measured at different tensile strains by using a Keithley 4200 semiconductor characterization system (Keithley Instruments, Cleveland, OH, USA). The electromechanical response of an InAs NW can be quantitatively described by the defined electromechanical gauge factor:

(4) $$GF=\frac{1}{\varepsilon }\frac{\Delta I}{I_{0}}$$

where *I*_0_ is the electrical current of the NW before stretching and *I* is the change of electrical current due to stretching. After the measurements in SEM, the tested NWs are placed onto the carbon film of a TEM grid through delicate nanomanipulations. Finally, the grid together with the NWs is transferred into a TEM for atomic-level determination of crystal structures (Figure 4d,e). Expect for the manipulator based tensile method, the electromechanical characterization of 1D nanostructures can also be realized with piezoelectric flexure stages [54] (Figure 5a) or microelectromechanical systems (MEMS) chips based tensile loading platform [48,55].

The piezoelectric properties of 1D nanomaterials can also be investigated with in-situ TEM method [56,57], where a STM-TEM probing system is generally used to manipulate the target NWs and measure the corresponding electrical properties. The STM-TEM probing system is generally composed of a piezo-tube driven movable end and a fixed end where free-standing NWs can be attached on. To form Ohmic contact, a focused ion beam and electron beam dual beam system can be used to precisely select, transfer and welded the NWs with the fixed end by Pt deposition during the sample preparation process [56] (Figure 5b). Then, the movable tungsten (W) tip is controlled to contact and deform the targeted NWs on the fixed end. During this deformation (bending, tension or compression) process, the current-voltage (*I-V*) curves under different mechanical strains can be simultaneously recorded (Figure 5c). Importantly, the crystal structure can also be analyzed by TEM characterization, providing an effective strategy to explore the crystal structure, orientation and defect dependent piezoelectric properties.

With above mentioned methods, the piezoelectric properties and mechanisms of 1D nanostructures can be systematically investigated. Firstly, the PFM method can be utilized to measure the piezoelectric coefficients, which are closely related to their energy conversion efficiency. This method can help to identify promising 1D nanomaterials for applications in NGs. When 1D materials with high piezoelectric coefficients are selected, their corresponding output voltage, power density and efficiency can be evaluated experimentally with the AFM lateral bending method. However, the PFM and AFM based methods can only be used to measure basic properties of 1D materials. To reveal the underlying mechanism and improve the performance, in-situ SEM/TEM methods can be further applied to study the influence of crystal structure, growth direction and defects on the piezoelectric properties of various kinds of 1D nanostructures from the view of microstructure, which will in turn provide instructions for the design and synthesis of 1D nanostructures. Then the piezoelectric properties of the newly designed 1D nanostructures with different chemical composition and microstructures will be re-evaluated with PFM and AFM based methods. Therefore, these methods are complementary to each other in the piezoelectric performance evaluation and material design of 1D nanostructures.

## 3. 1D Piezoelectric Materials

The enhanced piezoelectric effect, superior mechanical properties, and high sensitivity to small forces of 1D nanomaterials have made them promising candidates for PENGs. Until now, several kinds of 1D nanomaterials have been demonstrated to exhibited piezoelectricity mainly through the methods introduced in Section 2. In this section, piezoelectric properties and working mechanisms of various 1D nanostructures with WZ or zinc blend (ZB) structures, perovskite structures and 1D polymers will be introduced in detail.

### 3.1. Wurtzite or Zinc Blend Structured NWs

**Figure 6:**
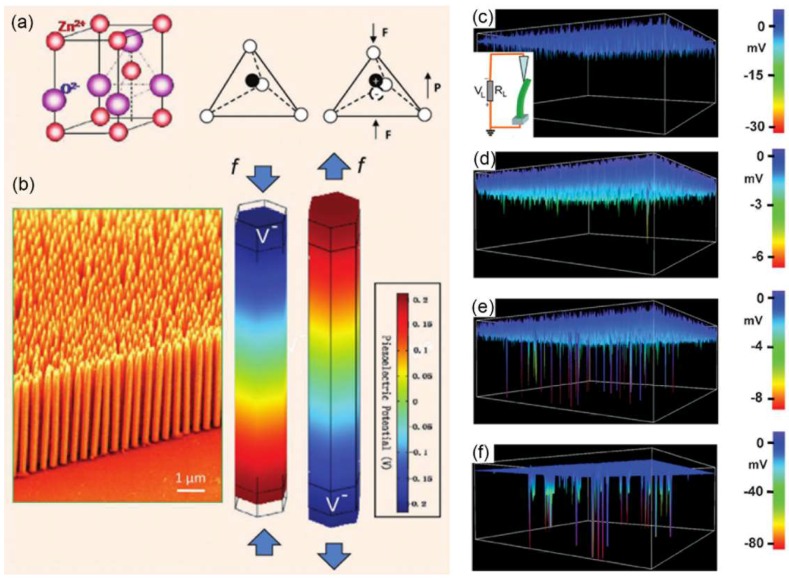
(**a**) Atomic model of wurtzite (WZ) ZnO and the compressive strain induced dipole moment; (**b**) Vertically aligned ZnO NW arrays and simulated piezoelectric potential along a ZnO NW under axial strain along the *c*-axis direction. Reproduced with permission from [59]. John Wiley and Sons, 2012. 3D electric signal images of (**c**) undoped AlN (10^−11^–10^−13^ Ω^−1^·cm^−1^); (**d**) Al_0.35_Ga_0.65_N (~0.5 Ω^−1^·cm^−1^); (**e**) GaN (~6–12 Ω^−1^·cm^−1^); (**f**) InN (~200–300 Ω^−1^·cm^−1^). Reproduced with permission from [46]. John Wiley and Sons, 2010.

Materials with WZ structure (shown in Figure 6a) possess a hexagonal structure with a large anisotropic property along the *c*-axis and its perpendicular direction. Its non-central symmetric structure can naturally lead to a piezoelectric effect when the material is mechanically strained. Under strain-free condition, centers of the tetrahedrally coordinated cations and anions in WZ crystals overlap with each other [58], thus WZ crystals show no polarization. When a strain is applied at an apex of the tetrahedron, the center of the cations and the center of the anions will be relatively displaced, resulting in a dipole moment. A constructive adds up of the dipole moments created by all of the units in the crystal will lead to a macroscopic potential drop along the straining direction in the crystal (Figure 6b). Therefore, a piezoelectric potential will be created in WZ materials. Both ZB and WZ have non-centrosymmetric structures, hence exhibiting piezoelectricity when subjected to strains along suitable directions.

Semiconducting materials in group II–VI and III–V generally possess WZ or ZB structure and are well known for their excellent optical and electronic properties [60,61]. They have been widely utilized for the applications of electronic and optoelectronic devices and their piezoelectric properties have been recently investigated. With the AFM lateral bending method, Wang and Song demonstrated the first ZnO NW arrays-based PENG with output voltage peaks of ~6 to 9 mV and output power density of ~10 pW/μm^2^ in 2006 [5]. Following this, a variety of other WZ structured NWs have been studied for energy harvesting application. As another group II–VI piezoelectric semiconducting material, the output signal of WZ ZnS NW is found to be significantly lower (~2 mV) than ZnO NW because of their smaller piezoelectric constants [62]. Lin et al. found that the single crystalline <0001> oriented WZ structured CdS NW (100 nm in diameter, >1 μm in length) exhibited a voltage output of around −3 mV [21], demonstrating that CdS NWs are also promising candidate for future nanoscale power devices. Except for the WZ structured materials, Hou et al. found that the lateral packaged single CdTe microwire (>500 μm in length, 1~2 μm in diameter) with mixed WZ/ZB phases can generate up to 0.3 V when strain is applied [63].

For 1D group-III nitride nanomaterials, Wang et al. have demonstrated increasing electricity generation in AlN nanocones, AlGaN nanocones (4 mV), GaN nanorods (7 mV) and InN nanocones (60 mV) with [0001] growth direction (Figure 6c–f), which is caused by the increasing piezoelectric potential and carrier density [46]. Subsequently, the highest output negative voltage of the individual InN NW (<5 μm in length, 25–100 nm in diameter) grown along $\left[10\bar{1}0\right]$ direction could reach −1 V and the corresponding NG shown excellent stability and reproducibility [22]. For n-type and p-type GaN NWs, the highest output voltage of single NW and the output power density of corresponding NGs could reach −300 mV, 12.5 mV/m^2^ and −350 mV, 12.7 mV/cm^3^, respectively [47,64]. Recently, the electric current generation has also been observed in individual WZ GaAs and InAs NWs [24,45].

Though the piezoelectric coefficients of these 1D WZ structured materials are much lower than that of perovskite materials or polymers, their unique advantage lies in the coupling between the piezoelectric and semiconducting properties. The piezoelectric properties will further extend their applications in FETs and improve the performance of various optoelectrical devices. The related applications will be discussed in Section 5.

### 3.2. Perovskite NWs/Nanofibers/Nanorods/Microbelts

**Figure 7:**
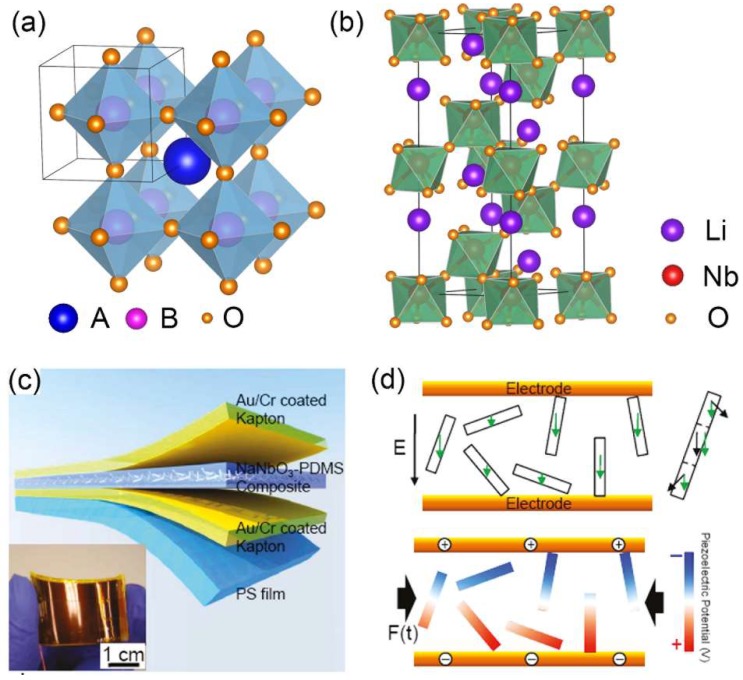
Atomic model of (**a**) cubic perovskite structure and (**b**) rhombohedral LiNbO_3_ structure; (**c**) Schematic diagram of the NaNbO_3_ based flexible NG and (**d**) its power generation mechanism. Reproduced with permission from [71]. American Chemical Society, 2011.

Considering the relatively low piezoelectric constant of above semiconductor NWs, the perovskite piezoelectric materials with high piezoelectric constant are quite desirable for energy harvesting. Adopted by many oxides [65], the perovskite structured materials have the chemical formula ABO_3_. As shown in Figure 7a, the perovskite structure consists of corner-sharing oxygen-octahedra with B cation in the center, and with A cation in the 12-coordinated position between 8 octahedra. However, the relative ion size requirements for stability of the cubic structure are quite stringent, so slight buckling and distortion can produce several lower symmetry distorted versions, in which the coordination numbers of A cations, B cations or both are reduced. The resulted non-centrosymmetric structure will finally lead to the piezoelectric properties of perovskite materials.

Lead zirconate titanate (PZT)-based ceramics are known for their excellent piezoelectric properties and have been widely used as actuators and sensors. Though bulk PZT possesses a high piezoelectric coefficient, mechanical failure can happen during their applications. As promising candidates for integrated nanosystems, 1D PZT nanostructures with excellent mechanical properties have been developed. Chen et al. have reported a piezoelectric NG based on high aspect ratio PZT nanofibers [66]. The lateral PZT nanofibers were connected to interdigitated electrodes and produced an 1.63 V output voltage when strain is applied. By using the low temperature grown vertical PZT NW array, Xu et al. reported a NG with an output voltage of 0.7 V and an average power density of 2.8 mV/cm^3^ [67], which is further used to light up a commercial LED. Since the piezoelectric coupling coefficient of single crystalline bulk (1 − *x*)Pb(Mg_1/3_Nb_2/3_)O_3_−*x*PbTiO_3_ (PMN−PT) is almost 30 times higher than that of BaTiO_3_ and almost 4 times higher than that of PZT bulk materials, lots of effort has been paid on synthesis of their 1D nanostructures [39,68]. With the hydrothermal process, Xu et al. successfully synthesized the 0.72Pb(Mg_1/3_Nb_2/3_)O_3_−0.28PbTiO_3_ NW and its piezoelectric coefficient has reached an average value of 373 ± 5 pm/V [69], which is 3 times larger than the highest reported value of 1D PZT nanostructures. They also fabricated a novel piezoelectric nanocomposite based on hierarchical PMN-PT NWs and a maximum output voltage of 7.8 V was obtained from the corresponding NG [70].

Despite the success in PZT due to their high polarization and piezoelectric performance as transducers, more attention has been paid on synthesis and investigation of lead-free materials which are more environmentally friendly. Except for the widely studied BaTiO_3_ NWs [54], the application of LiNbO_3_ type (LN type) structure which is similar to perovskite structure has also been explored. They possess a rhombohedral unit cell with a structure composed of oxygen octahedra containing Nb atom and surrounded by Li atoms (Figure 7b). Compared to the perovskite structure, the oxygen octahedra have been rotated around <111>, such that A atoms only have 6 oxygen first neighbors, rather than 12 as in the cubic perovskite structure [72]. With PFM method, the effective piezoelectric coefficient of LiNbO_3_ has been measured to be ~100 pm/V [37]. Additionally, a flexible NG with NaNbO_3_ NW mixed with PDMS composite as source of piezoelectric potential has been fabricated (Figure 7c) [73]. Due to its ferroelectricity, the piezoelectric domains of the randomly distributed NaNbO_3_ NWs can be effectively poled to one direction by high electric field (Figure 7d). Therefore, when strain is applied to this NG, the strain-induced electric polarization will also align to the dipole direction. This NaNbO_3_ based NG has shown a stable and high output piezoelectric signal with an open circuit voltage of 3.2 V and a power density of 0.6 mW/cm^3^ under a compressive strain of 0.23% [71]. Based on the large polarization in ZnSnO_3_ along the *c*-axis, ZnSnO_3_ is also one of the highly promising materials for lead-free piezoelectric energy harvesting and the output voltage and current of a ZnSnO_3_ microbelt based NG have been demonstrated to be 100 mV and 30 nA [74].

Additionally, the vertically grown (K_0.6_Na_0.4_)NbO_3_ nanorod array was found to exhibit a high piezoelectric coefficient of 180 pm/V and the corresponding NG can generate a stable high power density of ~101 μW/cm^3^, which is much higher than a BaTiO_3_ NW-based energy harvester (6.27 μW/cm^3^). Importantly, considering the ease in fine tuning the mole ratio of K/Na, the crystalline direction and Curie temperature of the nanorods, the above mentioned (K,Na)NbO_3_ nanorod array-based NG has great potential for high output power generation under harsh environment conditions, with a wide temperature range [75]. Therefore, the 1D perovskite structured materials not only exhibit an enhanced mechanical stability compared to their bulk counterparts, they also exhibit relatively larger piezoelectric coefficients than above mentioned WZ structured materials. Most importantly, their piezoelectric properties can be further improved by chemical composition and phase boundaries (see Section 4) etc., leading to a wider application in different environment.

### 3.3. 1D Polymers NWs/Nanofibers

**Figure 8:**
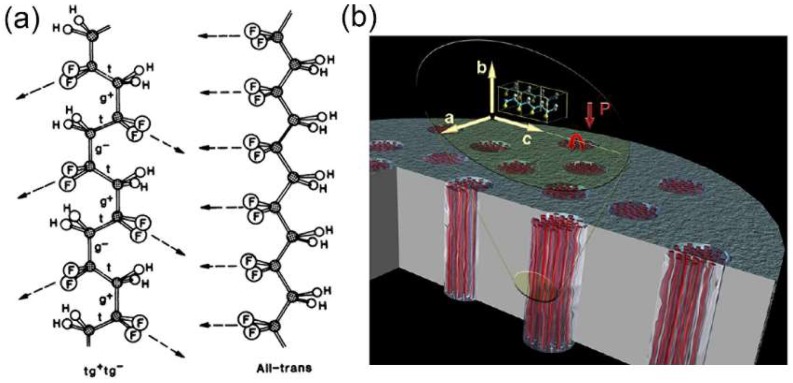
Schematic depiction of (**a**) α-phase (left) and *β*-phase (right) of the crystalline chain conformation of PVDF. The arrows indicate projections of the -CF_2_ dipole direction on planes defined by the carbon backbone. Reproduced with permission from [76]. the American Association for the Advancement of Science, 1983; (**b**) Scheme diagram of the molecular orientation of the polymeric chain templated in the mesoporous host. Both the *a*- and *c*-axes are in-plane with the alumina surface and the b-axis, as well as the polarization axis P, are aligned with the long axis of the NWs. Reproduced with permission from [80]. American Chemical Society, 2012.

Caused by the spatial arrangement of the chain segments in the crystalline phase, poly(vinylidene difluoride) (PVDF) and its copolymers possess intrinsic permanent dipole moments. Being lead free and biocompatible, they are also promising energy harvesting materials with advantages of flexibility, robustness, low weight and cost. The molecular formula of PVDF is (CH_2_–CF_2_)*_n_* and it can exist in five different crystalline forms [76], *α*, *β*, *γ*, *δ* and *ε*, while the most highly polar phase of PVDF is the *β*-phase, whose unit cell consists of two all-trans chains packed with their dipoles pointing in the same direction (Figure 8a). Therefore, these semi-crystalline piezoelectric polymers have attracted tremendous research interest. Typically, PVDF needs to be electrically poled (using an electric field of the order of 100 MV/m) or mechanically stretched to achieve the polar *β*-phase that shows the strongest piezoelectric behavior. The scalable template-wetting method can be used to grow aligned piezoelectric polymer NWs and the template-induced space confinement can promote the high crystallinity and preferential orientation of the lamellar crystals in the polymer NWs [77,78]. Though piezoelectric polymers show reduced piezoelectric properties compared with piezoelectric ceramics, it has been shown that geometrical confinement can have a profound influence on the final piezoelectric performances of these micromolecules [79]. Cauda et al. firstly observed the remarkable piezoelectric behavior of the PVDF NWs and found that nanoconfinement plays a crucial role in the enhancement of their final piezoelectric properties (Figure 8b) [80,81].

P(VDF–TrFE) [(CH_2_–CF_2_)*_n_*–(CHF–CF_2_)*_m_*] is more attractive since it crystallizes more easily into the *β*-phase due to the steric factors. With the template-wetting method, Whiter et al. have fabricated a P(VDF–TrFE) NW array based nanogenerator with a peak output voltage of 3 V at 5.5 nA in response to low-level vibrations [82]. By electrospinning onto a fast-rotating collector, large area and flexible sheets with aligned P(VDF–TrFE) nanofibers have been fabricated with excellent mechanical properties. Under bending conditions, these nanofibers can exhibit current up to 40 nA and voltage ~1.5 V and show high sensitivity in the low-pressure regime (0.1 Pa) [83]. Importantly, the voltage output of P(VDF–TrFE) fibers is found to closely related with the array density. The enhancement of the piezoelectric response in dense arrays is associated to the cooperative electromechanical effects [84]. Though research in piezoelectric polymers has been mainly focus on PVDF and its copolymers, their low Curie and/or melting temperatures have limited their applications at high temperature.

Due to the high degree of hydrogen bonding and dipole orientation resulting from the arrangement of polyamide molecules within adjacent chains upon crystallization, odd-numbered Nylons with relatively high melting temperatures generally possess ferroelectric and piezoelectric properties. Among odd-numbered Nylons, Nylon–11 (polyamide–11 = [C_11_H_21_ON]*_n_*) exhibits piezoelectric and ferroelectric properties comparable to PVDF at room temperature [85]. Recently, Datta et al. have firstly reported the fabrication of vertically aligned Nylon–11 NWs arrays with high crystallinity and intense orientation of the piezoelectric *γ*-phase by capillary wetting technique. The corresponding Nylon–11 NW array based NG can produce an open-circuit voltage of 1 V and short-circuit current of 100 nA when subjected to small-amplitude and low-frequency vibrations [86]. Importantly, this NG showed a stable performance at temperature as high as 150 ℃ which further expands the working temperature range of piezoelectric polymers.

To present a clear comparison about the piezoelectric properties of above mentioned 1D nanostructures, we have concluded their piezoelectric constants (*d*_33_) and the output voltage (*V*_output_) of corresponding NGs in Table 1 and Table 2, respectively. In short, for the reported 1D nanostructures, the WZ/ZB structured piezoelectric NWs show their advantages in the coupling of piezoelectric and semiconducting properties, exhibiting extended applications in piezotronic and piezo-photoronic devices. 1D perovskite structured nanomaterials generally possess much larger piezoelectric coefficients which can be further modulated with composition and microstructures, they are promising candidate in the field of NGs which require high output voltage and stabilities. Though exhibit weaker piezoelectric properties than perovskite ceramics, 1D polymers possess advantages of mechanically stable, chemically robust, cost effective and possibly biocompatible. These 1D piezo-materials provide different selections for the development of NGs in various fields and environments.

## 4. Performance Optimization

When materials’ size goes down to the nanoscale, various physical properties will be affected due to the size effect and large surface-to-volume ratio. In addition, different synthesis conditions and methods of 1D nanostructures will generally result in different phases, orientations and defects. Therefore, it is of vital importance to reveal the influence of these effects on the piezoelectric properties of 1D nanostructures, which will be discussed in this section.

### 4.1. Size Effect of Piezoelectric NWs

Due to the surface stiffening effect and quantum confinement effect, the mechanical and electrical properties of 1D nanostructured materials can be greatly influenced by diameters [48,90,91,92]. Similarly, the morphological design of 1D nanostructures is also quite important for the performance optimization of corresponding NGs.

**Figure 9:**
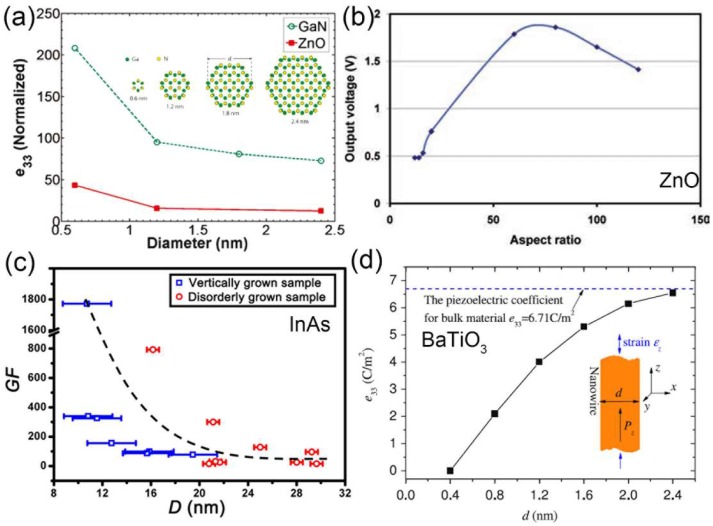
(**a**) The piezoelectric coefficient of GaN and ZnO NWs as a function of their diameter. Reproduced with permission from [94]. American Chemical Society, 2011; (**b**) The output electrical potential vs. aspect ratio of ZnO NWs controlled at constant diameter of 50 nm and changing the NW length from 600 to 6000 nm. Reproduced with permission from [95]. John Wiley and Sons, 2011; (**c**) The GF of InAs NWs with different diameters. Reproduced with permission from [24]. John Wiley and Sons, 2015; (**d**) The size-dependent piezoelectric coefficient of BaTiO_3_ NW. Reproduced with permission from [96]. IOP Publishing Ltd., 2010.

To reveal the piezoelectric size effect of 1D nanostructures, various theoretical calculations have been carried out. Due to the local changes in polarization and the reduction of unit cell volume, a giant piezoelectric size effect has been predicted in WZ ZnO and GaN NWs [93]. Their piezoelectric coefficients can be improved by two orders of magnitude if the NW diameter is reduced to less than 1 nm (Figure 9a) [94]. Additionally, the output voltage of ZnO NWs with different aspect ratios has been calculated with finite element method. At constant diameter of 50 nm, it has been found that the output voltage increases with the aspect ratio and starts to decrease when the aspect ratio reaches 80 (Figure 9b) [95]. Due to the difficulties in manipulation and the simultaneous measurement of electrical and mechanical performance of individual NWs with various diameters, the experimental explanation of the piezoelectric size effect of 1D nanostructures is quite challenging. By taking advantages of in-situ microscopy technique, the electromechanical properties of InAs NWs have been investigated and shown a rough trend that thinner InAs NWs possess larger gauge factor (Figure 9c) [24].

The surface tension plays an important role in the ferroelectric properties when the size of perovskite NWs decreases [97]. By taking into account this surface tension effect, it has been shown that Curie temperature (*T*_c_), mean polarization, area of hysteresis loop, coercive electric field and remnant polarization will decrease with the reduction of NW diameters [98,99,100]. In addition, calculations based on the Landau-Ginzburg-Devonshire theory indicated that the piezoelectric behaviors of PZT NWs will be enhanced when their diameter decreases [99]. This enhancement is caused by the decrease of *T*_c_, leading to the dielectric constant increases. Since the piezoelectric constant is proportional to the dielectric constant, the increase of the dielectric constant will result in the increase of the piezoelectric properties. Nonnenmann et al. investigated the piezoelectric response of the Au/PZT core/shell NWs with different thickness and radii by the aforementioned PFM method [101]. They found that the piezoelectric response of the 1D PZT nanoshell in the radial direction is much higher than the corresponding PZT thin film with the same thickness. With modified Landau-Ginzburg model, they found that the geometric curvature-driven polarization gradients in ultrathin films can lead to significant increase in *T*_c_, in contrast to the expected scaling of a depression of *T*_c_ with inverse thickness [101]. With the molecular dynamics method, Zhang et al. found that the piezoelectric coefficient of BaTiO_3_ NW increases with the diameter increase and approaches its counterpart bulk material when the diameter reaches 2.4 nm (Figure 9d) [96].

As we have mentioned before, NWs of piezoelectric polymers have been found to exhibit superior piezoelectric performance compared to films or bulk due to the nanoscale confinement effect [79]. Recently, vertically aligned P(VDF–TrFE) nanotube array with crystallographic polar axes oriented along the nanotube long axes has been fabricated with the anodized alumina membrane template. Accompanied with this preferred crystal and polarization orientation, the obtained piezoelectric coefficient is significantly higher (−35 pm/V) than that of monolithic film on substrate (−17.8 pm/V) [78]. Similarly, Datta et al. have firstly reported vertically aligned Nylon–11 NW array with high crystallinity by the capillary wetting template method. They found that the template-protected NWs show more ordered α phase and less *γ* phase, while partial recrystallization from *α* to *γ* can occur under mechanical deformation during template dissolution in the freed NWs, resulting in intense orientation of the piezoelectric *γ* phase and exhibiting the self-poled nature [86]. Therefore, by virtue of the nanoconfinement effect, 1D nanostructures of piezoelectric polymers generally possess remarkable piezoelectric properties.

It should be noted that the piezoelectric properties of different 1D nanomaterials generally follow different size effect and the optimized diameter and orientation should be selected accordingly.

### 4.2. Crystal Structure and Orientation Dependent Piezoelectric Properties of NWs

To figure out the crystal structure exhibiting maximum piezoelectric property, it is quite necessary to experimentally establish the relationship between piezoelectric properties of NWs and their crystal structures. As we have discussed, the piezoelectric performance of 1D polymers relies greatly on their crystal phases. Among the five semi-crystalline polymorphs (*α*, *β*, *γ*, *δ* and *ε*) of PVDF, the *α*, *β* and *γ* phases are the most investigated and used. The *α* phase is non-polar, while the polar *β* and *γ* phases display piezoelectricity. Through the modification of the template nanopore surface with oxygen plasma and aminopropyltrimethoxysilane (APMS), the crystallization of the PVDF electroactive phase (*β* and *γ* phases) can be enhanced by the surface-induction nucleation effect [102]. Strategies like electrically poling and/or mechanical stretching are generally applied to orient the molecular dipoles in the same direction in order to induce the transformation into *β* phase. However, it has been reported that the preferential crystallization of PVDF NWs into the *β* phase with *b*-axis along their axial direction can also be realized through nanoconfinement in absence of poling or stretching (Figure 8b) [80,81], resulting in remarkable piezoelectric behavior. The nanoconfinement plays a crucial role in the enhancement of the final piezoelectric features of the templated NWs. Similarly, assisted by the geometrical confinement, the *β*-phase P(VDF-TrFE) NWs with *a*-axis along their long axes have also been fabricated and the improved piezoelectric coefficient has been obtained.

**Figure 10:**
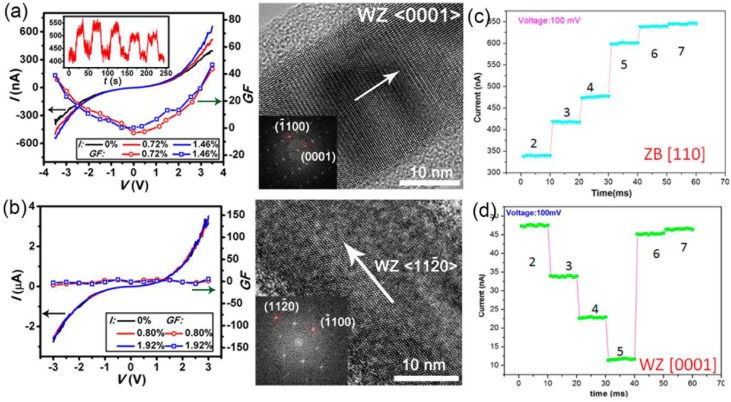
*I*-*V*, *GF*-*V* curves and corresponding high resolution TEM images of (**a**) <0001> orientated WZ InAs NW with stacking faults and (**b**) single-crystalline $<11\bar{2}0>$ oriented WZ InAs NW at different axial tensile strains. Reproduced with permission from [24]. John Wiley and Sons, 2015. The variation of the electrical current with increasing deformation of (**c**) a <110> orientated zinc blend (ZB) InAs NW and (**d**) a <0001> orientated WZ InAs NW. Reproduced with permission from [56]. American Chemical Society, 2016.

The piezoelectric matrix of NWs with WZ structure consists of three independent coefficients (*d*_33_, *d*_13_, and *d*_15_) and can be investigated with the aforementioned PFM method. It has been reported that these three independent piezoelectric coefficients of the WZ GaN NWs grown along [0001] direction are measured to be 12.8, −8.2 and −10.2 pm/V (Figure 2), respectively [23]. Similarly, the average value of *d*_33_ and *d*_31_ for the BiTiO_3_ coated carbon fibers are measured to be 31.6 ± 14.5 pm/V and −5.4 ± 3.2 pm/V, respectively [43]. To investigate the influence of crystal structure and growth direction on the electromechanical properties of NWs, we have studied the electromechanical properties of individual WZ InAs NWs grown along different crystallographic directions with aforementioned in-situ SEM technique (Figure 4) and determined the crystal structures of the studied NWs by TEM. With this method, we found that the piezoelectric and piezoresistive effects of InAs NWs strongly depend on the crystallographic directions along NW axis with the maximum effects along WZ <0001> directions (Figure 4b, Figure 10a). Additionally, negligible electromechanical response of the WZ NWs grown along $<11\bar{2}0>$ directions (Figure 10b), and ZB NWs grown along <011>, <103>, and $<2\bar{1}\bar{1}>$ directions up to a strain of ~2% indicate that InAs NWs exhibit negligible piezoelectric and piezoresistive effects along these directions [24]. Moreover, Zhang et al. have investigated the electromechanical responses of the defect-free ZB and WZ-structured InAs NWs with different orientations by in-situ TEM techniques [56]. By forming Ohmic contacts between NWs and electrodes, they found that the conductance of the defect-free ZB structured <110> and WZ-structured <0001> InAs NWs increases and decreases respectively under compressive deformation processes (Figure 10c,d). Therefore, expect for the crystal-structure-dependent piezoelectric properties of InAs NWs, their piezoresistive effect are also crystallization orientation dependent [56]. Boxberg et al. found that the piezoelectric field induced by lattice mismatch in InAs/InP NW heterostructures is generally much stronger in WZ NWs than in its corresponding ZB NWs [103,104].

### 4.3. Influence of Doping on NW-Based NGs

It has been widely investigated that the electrical properties can be greatly influenced by chemical doping, phase mixing and stacking faults [105,106,107]. It has been shown that chemical doping can also be used as a compatible strategy for realizing high performance energy-harvesting devices by lattice strain or supression of the undesirable screening effect.

**Figure 11:**
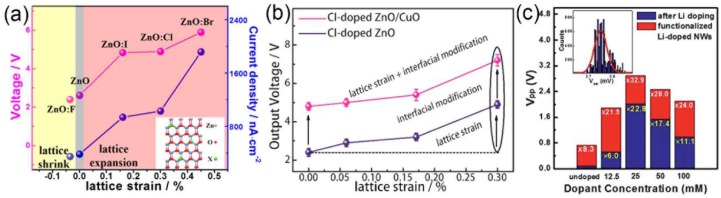
(**a**) The dependence of the output performance of ZnO piezoelectric NG on the lattice strain induced by different halogen dopant. Reproduced with permission from [108]. American Chemical Society, 2015; (**b**) Output performance of ZnO NW film NG as a function of the doping concentration of Cl. Reproduced with permission from [114]. American Chemical Society, 2016; (**c**) Histogram of piezoelectric output voltages for undoped and Li-doped ZnO NW samples and the influence of surface functionalization. Reproduced with permission from [113]. The Royal Society of Chemistry, 2013.

Given the ionic size difference between dopants and oxygen, Zhang et al. reported that the lattice strain along the ZnO NW *c*-axis can be tuned from compressive to tensile state by a substitution of halogen dopant (F, Cl, Br, I) [108]. The induced lattice strain will significantly facilitates the piezocharge separation under applied stress and can be applied to enhance the performance of ZnO NW array based NGs (Figure 11a). With this lattice strain strategy, Liu et al. found that the output voltage and current density of the Cl doped ZnO NWs can be enhanced with the increase of dopant concentration (Figure 11b). The piezoelectric coefficient of ternary CdS*_x_*Se_1−*x*_ NW can also be tuned by the composition and the piezo-phototronic effect was stronger as the ratio of S/Se increases [109]. Therefore, the selection of dopant and their concentrations are important matters for the piezoelectric performance of ZnO NWs. It should be mentioned that the large ionic size and high doping concentration of the dopant may also lead to more lattice defects, thus increasing the concentration of free electrons and decreasing the piezoelectric output of ZnO NW based NGs.

Since the piezoelectric characteristic of semiconducting NW is significantly influenced by the free charge carriers, the controlment of the charge carrier density is very important for the performance optimization of NGs. When considering the competition between the reduction of inner resistance and the screening effect on piezoelectric potential, an optimum carrier concentration should be selected for the maximization of the output performance of NG. For Si-doped GaN NW based NGs, the output current of individual NW increases with doping concentration and reaches a maximum output current of ~50 nA at 5.63 × 10^18^ cm^−3^. The output current is subsequently decreased with further increase in the carrier concentration [110]. Generally, a high carrier concentration can lead to a more pronounced screening effect, thus leading to a pronounced reduction in the piezopotential [111,112]. Due to the oxygen deficiencies, the as grown ZnO NW is naturally n-type semiconducting and p-type doping can be utilized to reduce the screening effect [111]. Sohn et al. performed piezopotential measurements on a Li-doped ZnO NW array based NG with dopant concentration ranging from 0 to 100 mM [113]. As shown in Figure 11c, the output voltage is correspondingly enhanced with Li concentration up to 25 mM because of a continuous decrease in donor concentration, while gradually reduced with further increasing Li concentration due to the compensation effect like formation of acceptor complexes, deep levels etc. [113].

In addition, the output performance of ZnO based NGs can be further improved through the deposition of p-type materials like CuO and NiO. By forming a p-n heterojunction, a depletion region and built-in electric field near the interface can effectively deplete the free electron in the n-type ZnO NW array layer, thus reducing the screening effects and leading to an enhancement of output voltage [114,115]. With this approach, the maximum output voltage is measured to be ~7.5 V, which is about 7-fold higher than ZnO NG without a CuO layer [115]. The output voltage of a NiO/ZnO based NG is up to 430 mV, which is about 21-fold higher than that of the pristine ZnO NG [116].

### 4.4. Influence of Phase Bounday on the Piezoelectric Properties of NWs

Our previous work revealed that the piezoelectric properties of InAs NWs can be greatly suppressed by the presence of stacking faults in them [24]. The stacking faults in WZ InAs NWs can be treated as small segments of ZB structures. Because the bandgap of WZ is larger than that of ZB phases, the stacking faults can act as quantum wells for electrons and potential barriers for holes, resulting in the separation of electrons and holes, and thus a charge polarization at WZ/ZB interfaces, which may counteract the piezoelectric field generated by the strained WZ segments. Therefore, the total piezoelectric effect of the whole NW is suppressed (Figure 4b, Figure 10a). Since the ZB phase is non-piezoactive, CdS NWs with alternating WZ and ZB phases along the growth direction exhibit a low voltage (0.5~1 mV) output compared with the pure WZ phase CdS NWs (3 mV) [21].

**Figure 12:**
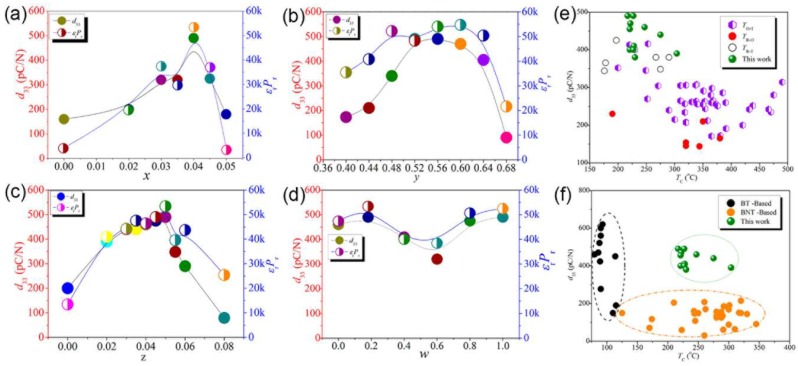
The change of *d*_33_ with chemical composition of (1 − *x*)(K_1−*y*_Na*_y_*)(Nb_1−*z*_Sb*_z_*)O_3−*x*_Bi_0.5_(Na_1−*w*_K*_w_*)_0.5_ZrO_3_ at (**a**) *y* = 0.52, *z* = 0.05, *w* = 0.18; (**b**) *x* = 0.04, *z* = 0.05, *w* = 0.18; (**c**) *x* = 0.04, *y* = 0.52, *w* = 0.18; (**d**) *x* = 0.04, *y* = 0.52, *z* = 0.05. *d*_33_ as a function of T_C_ for (**e**) KNN-based piezoceramics and the (1 − *x*)(K_1−*y*_Na*_y_*)(Nb_1−*z*_Sb*_z_*)O_3−*x*_Bi_0.5_(Na_1−*w*_K*_w_*)_0.5_ZrO_3_ ceramics and (**f**) Bi_0.5_Na_0.5_TiO_3_ (BNT)- and BaTiO_3_ (BT)-based piezoceramics and the (1 − *x*)(K_1−*y*_Na*_y_*)(Nb_1−*z*_Sb*_z_*)O_3−*x*_Bi_0.5_(Na_1−*w*_K*_w_*)_0.5_ZrO_3_ ceramics. Reproduced with permission from [119]. American Chemical Society, 2014.

The composition phase diagrams of piezoelectric perovskite ceramics can display a transition region known as a morphotropic phase boundary (MPB), where the crystal structures can change from the tetragonal (T) phase to rhombohedral (R) phase. This MPB region from T phase to R phase is found to mediated by the monoclinic phase [117]. Due to the enhanced polarizability induced by the coupling between two equivalent energy states of the T and R phases, the MPB region generally possesses excellent piezoelectric properties [118]. For the piezoelectric properties of lead-free perovskite ceramics, great efforts have been given on the phase transition of potassium-sodium niobate (KNN)-based ceramics by finely tailoring their composition. To further enhance *d*_33_ of KNN ceramics, Wang et al. have constructed a R-T phase boundary in (1 − *x*)(K_1−*y*_Na*_y_*)(Nb_1−*z*_Sb*_z_*)O_3−*x*_Bi_0.5_(Na_1−*w*_K*_w_*)_0.5_ZrO_3_ by optimization of the chemical composition (Figure 12a) [119]. Specifically, the peak *d*_33_ of 490 pC/N was obtained with *x* = 0.04, *y* = 0.52 and *w* = 0.18, which is superior to other results on KNN-based ceramics (Figure 12b) and is comparable to some of those of PZT-based ceramics (Figure 12c). Similarly, Zhang’s and Rubio-Marcos’s groups have also attained a large *d*_33_ of >400 pC/N by the modification of the R-T phase boundaries and domain structures of KNN-based materials [120,121]. Since the coexistence of two phases can provide more polarization, Meng et al. have recently synthesized orthorhombic KNN NWs and perovskite KNN NWs with MPB regions by hydrothermal method. They found that the (K_1−*x*_Na*_x_*)NbO_3_ NWs with a morphotropic R–T phase boundary exhibit a higher piezoelectric coefficient (28.93 pm/V) than that of KNN NWs with single orthorhombic structures (26.37 pm/V) [122]. Joung et al. synthesized orthorhombic (O) phase, T phase and O–T KNbO_3_ NWs with hydrothermal methods and measured *d*_33_ are 11.6, 23.5 and 40 pm/V, respectively [123].

As is well-known, the physical properties are generally determined by the microstructures of materials. With a better understanding of the effects of size, phase and defects, the piezoelectric properties can be successfully optimized according to their application environment and requirements.

## 5. Application of 1D Piezoelectric Materials

The piezoelectric 1D nanostructures have been widely demonstrated to harvest various kinds of mechanical energies and the detailed fabrication process, structures and working mechanisms have been systemically summarized in several other review papers [124,125,126]. Expect for their applications in NGs, the piezoelectric properties of 1D semiconducting nanostructures have been further expanded in the fields of piezotronics and piezo-phototronic [58].

When strain is applied on ZnO NWs, the induced piezopotential not only can drive a transient flow of electrons in the external load to serve as a nanogenerator for energy harvesting, but also can act as a gate voltage that controls the carrier flow through a ZnO NW based FET, in which the gate voltage is replaced by the piezopotential [127,128]. It has been reported that the effective potential barrier at the interface between ZnO NW channel and the dielectric layer can be varied by the strain induced piezoelectric potential and the electronic transport properties of ZnO NW FETs like current, transconductance, mobility and threshold voltage can be greatly influenced [129]. Importantly, hybrid FET composed of single-walled carbon nanotubes (SWNTs) or MoS_2_ flakes which serves as a carrier transport channel, and a ZnO piezoelectric fine wire (PFW) or vertically grown ZnO NW array acting as the power-free and contact-free gate has been constructed. The created piezopotential by external strain in the ZnO can control the charge transport in the SWNT or MoS_2_ channel located underneath [25,130]. Similarly, other piezoelectric NWs like GaN and PZT can also be utilized in this kind of piezopotential gated FETs.

For optoelectronic materials with piezoelectric properties, the strain induced piezoelectric potential can also be utilized to tune the Schottky barrier height or p-n junction built-in potential at the contact/interface. Therefore, the photon emission process can be effectively tuned by controlling the carrier generation, transport and recombination processes by the externally applied strain [131,132,133]. With this piezo-phototronic effect, the photodetection performance of a ZnO NW-based photodetector and the CdSe/ZnTe core/shell NW arrays have been dramatically enhanced under external load [20,134]. Importantly, by using a piezoelectric GaN NW as the local gate, the MoS_2_ FET exhibit fast photoswitching in 5 ms as a photodetector and the photoresponsivity can be further improved [135]. Due to the band modification caused by the piezopotential and the creation of a trapping channel for holes at the ZnO/GaN interface region, the emission intensity, injection current and conversion efficiency of a ZnO microwire-based light emitting diode have been enhanced under compressive strain [28]. Moreover, the performance of the photovoltaic (PV) devices can also be tuned by the piezo-phototronic effect. Pan et al. have firstly reported that the performance of the n-CdS/p-Cu_2_S core-shell NW PV have been enhanced by as high as 70% under strain [27]. Importantly, the piezo-phototronic effect can also be utilized to improve the efficiency of large-scale solar cells [136,137]. By utilizing the energy harvested from the environment to drive electronic devices, self-powered nanosystems consist of NGs and various electronic devices like photodetectors, light emitting diode, sensors etc. have been demonstrated [138,139,140].

## 6. Summary and Future Perspectives

In this review, we mainly focus on the characterization and optimization of the piezoelectric properties of 1D nanomaterials. The piezoelectric coefficients, performance of NW-based NGs and crystal structure-dependent electromechanical properties of 1D nanostructured materials can be comprehensively investigated through PFM method, AFM lateral bending and in-situ SEM/TEM methods, respectively. Based on these methods, the piezoelectric properties of semiconducting NWs, perovskite NWs and 1D polymers are subsequently introduced and compared. Furthermore, strategies for the piezoelectric performance improvement have been summarized from the view of microstructures, including size, crystal structure, orientation and defects. Finally, the extension of 1D piezoelectric materials in FET and optoelectronic devices are simply introduced. This review provides a clear understanding of 1D piezoelectric nanomaterials from material structure, characterization methods and performance improvement and connects the piezoelectric properties with the microstructure of 1D nanomaterials.

Most of the high quality WZ or ZB structured NW arrays are grown by the molecular beam epitaxy or chemical vapor deposition methods, which greatly increase the fabrication cost of corresponding NGs. Though 1D piezoelectric polymers possess much easier synthesis process (template-assisted, electrospinning or nanoimprinting), their working/melting temperatures and piezoelectric properties are relatively low. Therefore, efforts should be paid on exploring cost effective material synthesis processes which can realize the better control of their diameter, crystal orientation and chemical composition, and measures should be taken to further expand the working temperature of perovskite and piezoelectric polymers. Considering the advantages provided by different piezoelectric materials, 1D nanostructured composites/heterostructures consist of different piezoelectric materials may obtain combined advantages and their applications in NGs need to be explored. Moreover, how to integrate 1D nanostructure based NGs into self-powered systems to effectively drive electrical devices like FET, LED or photodetectors is also a critical step toward their practical applications and has attracted lots of research interest. Importantly, to increase the energy conversion ability of NGs, the development of hybrid nano-energy harvesting system which can convert different forms of energy (mechanical, heat, etc.) into electricity is also a trend in the future studies.

## Figures and Tables

**Table 1:** Summary of the piezoelectric constants (*d*_33_) of 1D WZ structured materials, perovskite materials and polymers.

Structure	Materials	*d*_33_ (pm/V)	References
WZ structure	ZnO	14.3–26.7 (Nanobelt)	[36]
	GaN	12.4 (NW)	[23]
	GaAs	26 (NW)	[45]
	CdS	10.32 (Bulk)	[87]
	InN	7.6 (Calculation)	[88]
	AlN	5.4 (Calculation)	[88]
Perovskite	PZT	127 (Fiber)	[89]
	PMN–PT	50 (Nanofiber)	[39]
		373 (NW)	[69]
	LiNbO_3_	100 (NW)	[37]
	(K,Na)NbO_3_	180 (Nanorod)	[75]
	BaTiO_3_	45 (NW)	[54]
Polymer	P(VDF–TrFe)	~35 (NW)	[78,82]
		25–45 (NW)	[77]
	PVDF	6.5 (NW)	[81]
		~10–20 (NW)	[80]
	Nylon–11	3–12 (Films, *d*_31_)	[86]

**Table 2:** Summary of output voltage (*V*_output_) of different 1D nanostructure based NGs. Their corresponding diameter *D*, length *L* and forms of NGs are also listed.

Structure	Materials	*V*_output_ (mV)	*D* (nm)	*L* (μm)	References	Forms of NGs
WZ	ZnO	~6–9 (NW)	~20–40	0.2–0.5	[5]	AFM lateral bending: Single NW
	ZnS	2 (NW)	~100	~2	[62]	
	CdS	~–3 (NW)	~100	>1	[21]	
	InN	60 (Nanocone) ~–1000 (NW)	~200–400 25–100	~1 ~5	[46] [22]	
	GaN	–300 (NW) 7 (Nanorod)	~50 ~500	~3–4 ~5	[47] [46]	
	CdTe	~300 (MW)	1000–2000	100–800	[63]	Lateral packed single NW NG
Perovskite	PZT	1630 (Nanofibers) 700 (NWs)	60 ~500	500 ~5	[66] [67]	PDMS packed lateral PZT nanofibers Vertical PZT NW arrays
	PMN–PT	9 (NW) 7800 (NWs)	~500 ~200–1000	~5 ~5–10	[68] [70]	Lateral packed single NW NG NWs-PDMS composite
	NaNbO_3_	3200 (NWs)	~200	~10	[71]	NWs-PDMS composite
	ZnSnO_3_	100 (Nanobelt)	100	1000	[74]	Lateral packed single nanobelt NG
Polymer	P(VDF–TrFe)	3000 (NWs) 1500 (Nanofibers)	~200 ~200	~60	[82] [83]	Vertical NW arrays Highly aligned nanofiber arrays
	Nylon–11	1000 (NWs)	~200	~40-50	[86]	Vertical NW arrays
